# Is there an association between enhanced choline and β-catenin pathway in breast cancer? A pilot study by MR Spectroscopy and ELISA

**DOI:** 10.1038/s41598-017-01459-z

**Published:** 2017-05-22

**Authors:** Khushbu Agarwal, Gururao Hariprasad, Komal Rani, Uma Sharma, Sandeep R. Mathur, Vurthaluru Seenu, Rajinder Parshad, Naranamangalam R. Jagannathan

**Affiliations:** 1 Department of NMR & MRI Facility, All India Institute of Medical Sciences, New Delhi, 110029 India; 2 Department of Biophysics, All India Institute of Medical Sciences, New Delhi, 110029 India; 3 Department of Pathology, All India Institute of Medical Sciences, New Delhi, 110029 India; 4 Department of Surgical Disciplines, All India Institute of Medical Sciences, New Delhi, 110029 India

## Abstract

Total choline (tCho) was documented as a biomarker for breast cancer diagnosis by *in vivo* MRS. To understand the molecular mechanisms behind elevated tCho in breast cancer, an association of tCho with β-catenin and cyclin D1 was evaluated. Hundred fractions from 20 malignant, 10 benign and 20 non-involved breast tissues were isolated. Cytosolic and nuclear expressions of β-catenin and cyclin D1 were estimated using ELISA. Higher tCho was seen in malignant compared to benign tissues. Malignant tissues showed higher cytosolic and nuclear β-catenin expressions than benign and non-involved tissues. Within malignant tissues, β-catenin and cyclin D1 expressions were higher in the nucleus than cytosol. Cyclin D1 expression was higher in the cytosolic fractions of benign and non-involved than malignant tissues. Furthermore, in malignant tissues, tCho showed a positive correlation with the cytosolic and nuclear expression of β-catenin and cyclin D1 and also a correlation between nuclear expressions of both these proteins was seen. Higher cytosolic β-catenin expression was seen in progesterone receptor negative than positive patients. Results provide an evidence of correlation between non-invasive biomarker, tCho and the Wnt/β-catenin pathway. The findings explain the molecular mechanism of tCho elevation which may facilitate exploration of additional therapeutic targets for breast cancer.

## Introduction

Breast cancer ranks second as a cause of cancer death in women (after lung cancer) and is a substantial cause of mortality and morbidity among women worldwide^1^. In 2015 the incidence rate of breast cancer in Indian women of all ages was 21%^2^. Steroid hormone and growth factors regulated pathways play an important role in maintaining the breast tissue architecture. Thus, a disruption of these pathways results in an altered cell-cell and cell-extracellular matrix interaction that eventually leads to uncontrolled cellular proliferation and development of a tumor^3^. Both estrogen and progesterone are examples of two such steroid hormones which are not only involved in the regulation of mammary gland development, but are also found to be associated with the initiation, development, and progression of breast cancer^3^. After binding to their respective intracellular receptors [Estrogen Receptor (ER) and Progesterone Receptor (PR)] both these hormones activate a set of signalling events that ultimately result in enhancing the cellular proliferation rate of normal breast epithelium^4^. ER−/PR− breast tumors are shown to have a poor prognosis compared to ER+/PR+ tumors^5^. Similarly, human epidermal growth factor (Her2neu) status is one of the other determinants of the rate of breast tumor proliferation, with Her2neu+ tumors representing a more aggressive form^6^. Currently, the treatment protocol adopted for breast cancer patients is based on the expression profiles of ER, PR, and Her2neu on human tumor cells^7^.

**Figure 1:**
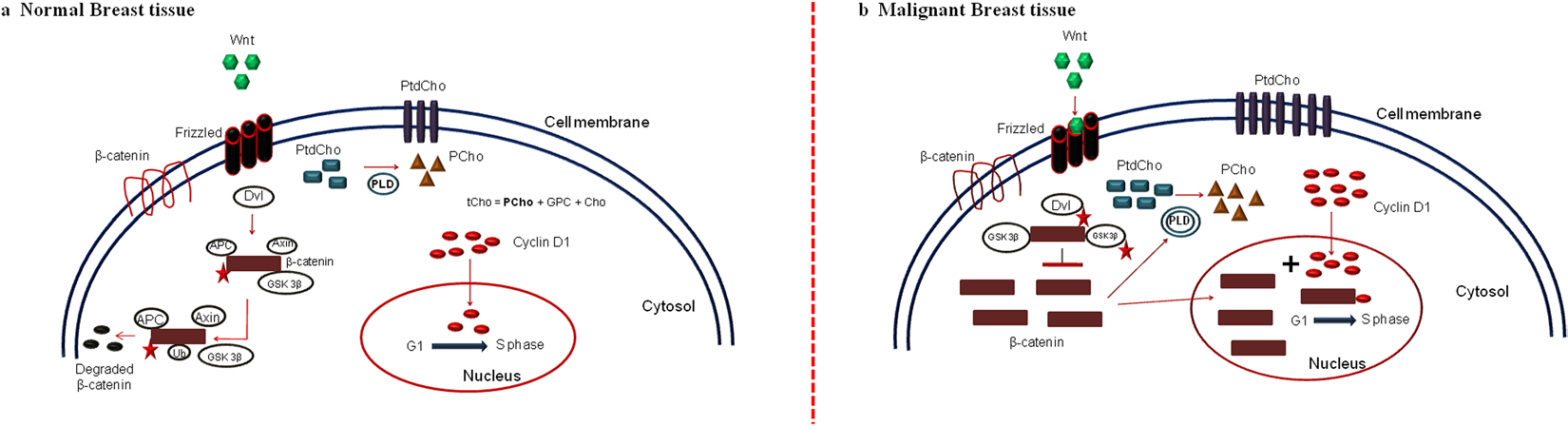
Schematic representation showing the link between the choline synthesis and the Wnt-mediated β-catenin pathway. (**a**) Normal breast tissue: In a normal breast tissue Wnt (green polygon) signalling is absent and β-catenin (brown rectangles) level is maintained low in the cell cytosol due to phosphorylation (red stars) and degradation of β-catenin. Also, cyclin D1 (red circles) translocates to the nucleus at a reduced rate to participate in G1 to S phase transition. The cell division rate is regulated. Phosphatidyl choline (PtdCho; blue rectangle) which is a membrane phospholipid, converts into phosphocholine (PCho; brown triangle) in the cytosol by the activity of phospholipase D (PLD; grey circle). (**b**) Malignant breast tissue: During malignancy, Wnt signalling is active and hence β-catenin increases in the cytosol which can translocate to the nucleus and bind to cyclin D1 (brown rectangle + red circle), to increase the rate of cellular transcription. With the increase in cellular proliferation, membrane requirement for PtdCho increases which in turn leads to increased PLD activity (double grey circle). PCho increases in the cell cytosol, thereby increasing the tCho levels. Increased cytosolic β-catenin levels also increases the activity of PLD.

Among the several molecular pathways involved in breast cancer development, Wnt/β-catenin pathway is best characterized^8^. β-catenin is a cellular adhesion protein which regulates the gene transcription rate^9^. The schematic representation of Wnt mediated β-catenin pathways in normal and malignant breast tissues is shown in Fig. 1. In normal tissue, Wnt signalling is inactive and β-catenin is maintained at low levels in the cytoplasm owing to its degradation via the ubiquitin-proteasome pathway. A multiprotein destruction complex is formed with the phosphorylation of β-catenin (at Serines 33, 37, and Threonine 41) by dishevelled protein (Dvl) and binding of Axin, adenomatous polyposis coli (APC), and glycogen synthase kinase 3 β (GSK 3β). This is followed by poly-ubiquitination and subsequent degradation of β-catenin by the ubiquitin-proteasome mediated pathway.

However during malignancy, Wnt ligand binds with frizzled (Fzd) receptor protein along with either lipoprotein receptor-related protein (LRP5) or LRP6, forming a ternary complex on the extracellular membrane. Consequently, phosphoprotein Dvl translocates to the ternary complex at the plasma membrane. This leads to translocation of Axin and GSK 3β (phosphorylated by Dvl) from the cytoplasm to the receptor complex. As a result, the destruction complex dissociates, increasing the cytoplasmic concentration of stabilized β-catenin. The accumulated β-catenin then translocates to the nucleus, binds to T-cell factors (TCF) and activates the transcription of cyclin D1 (located on human chromosome band 11q13)^8, 10, 11^.

Cyclin D1 encoded protein is expressed during the G1 phase and is essential for G1-S phase transition during the cell cycle. The protein binds and forms complexes with cyclin-dependent kinases (CDKs) and proliferating cell nuclear antigens, thereby regulating cellular proliferation^12^. The implication of cyclin D1 in various malignancies, including breast cancer^13, 14^ emphasizes the importance of studying this protein in malignant transformation and cancer progression.

*In vivo* proton (^1^H) MRS studies have consistently reported an elevation of total choline [(tCho; comprising of free choline, glycerophosphocholine (GPC) and phosphocholine (PC)] in human breast tumors^15–22^. Choline phospholipid metabolism is reported to be an important hallmark of cancer progression^15^. PC is a major contributor of tCho in cell membranes^23^ and its increase during malignancy implies an increase in the activities of enzymes involved in biosynthetic and catabolic pathways in cells^24^. This aberrant increase in membrane lipids correlates well with the increased cellular replication and membrane synthesis during malignancy^21, 25, 26^. Among the several oncogenic signalling pathways, RAS signalling is demonstrated to affect the activities of all enzymes involved in choline metabolism. The activity of CTP:phosphocholinecytidylyltransferase (CCT) is reported to be elevated by the activation of mitogen activated protein kinase (MAPK) pathway^27^. The activation of PtdCho specific-phospholipase D (PLD) is regulated by two positive feedback (mediated by Ral Guanine Nucleotide Dissociation Stimulator; RALGDS and Phosphoinositide 3-kinase; PI3K) and negative feedback (mediated by Rapidly Accelerated Fibrosarcoma; RAF and RALGDS) mechanisms during RAS signalling. Furthermore, stimulation with platelet derived growth factor (PDGF) increases the activity of PtdCho specific-phospholipase C (PLC) via RAF1^27^. PDGF is also demonstrated to increase the activities of PLD and choline kinase (CHK)^27^. While, c-Jun terminal kinases (JNK) and extracellular signal–regulated kinases (ERK) increases phospholipase A2 (PLA2) activity. Another signalling pathway i.e., PI3K–AKT and several transcription factors like JUN, MYC and HIF1 also regulate the activity of CHK. The activation of these enzymes consequently leads to elevated levels of PC in the malignant cell^27^. During breast malignancy, an increase in phosphocholine content is the result of enhanced expression and activities of several enzymes like CHK^24, 28^, PLC, PLD^24, 29^ and/or, choline transport^30^. Among these enzymes, previous studies have shown PLD to be co-expressed with β-catenin in human colorectal cancer^31^.

Thus, previous studies have indicated a major role for activated choline phospholipid metabolism^8^ and Wnt-mediated β-catenin signalling^15^ pathways in cancer progression. However, to the best of our knowledge, there is no literature report on the demonstration of the connection between these two pathways in breast cancer. Therefore, the present study investigated the link between these two major molecular pathways that may have important implications in devising new diagnostic strategies for breast cancer patients.

## Results

### Total choline concentration in different breast tissues

**Figure 2:**
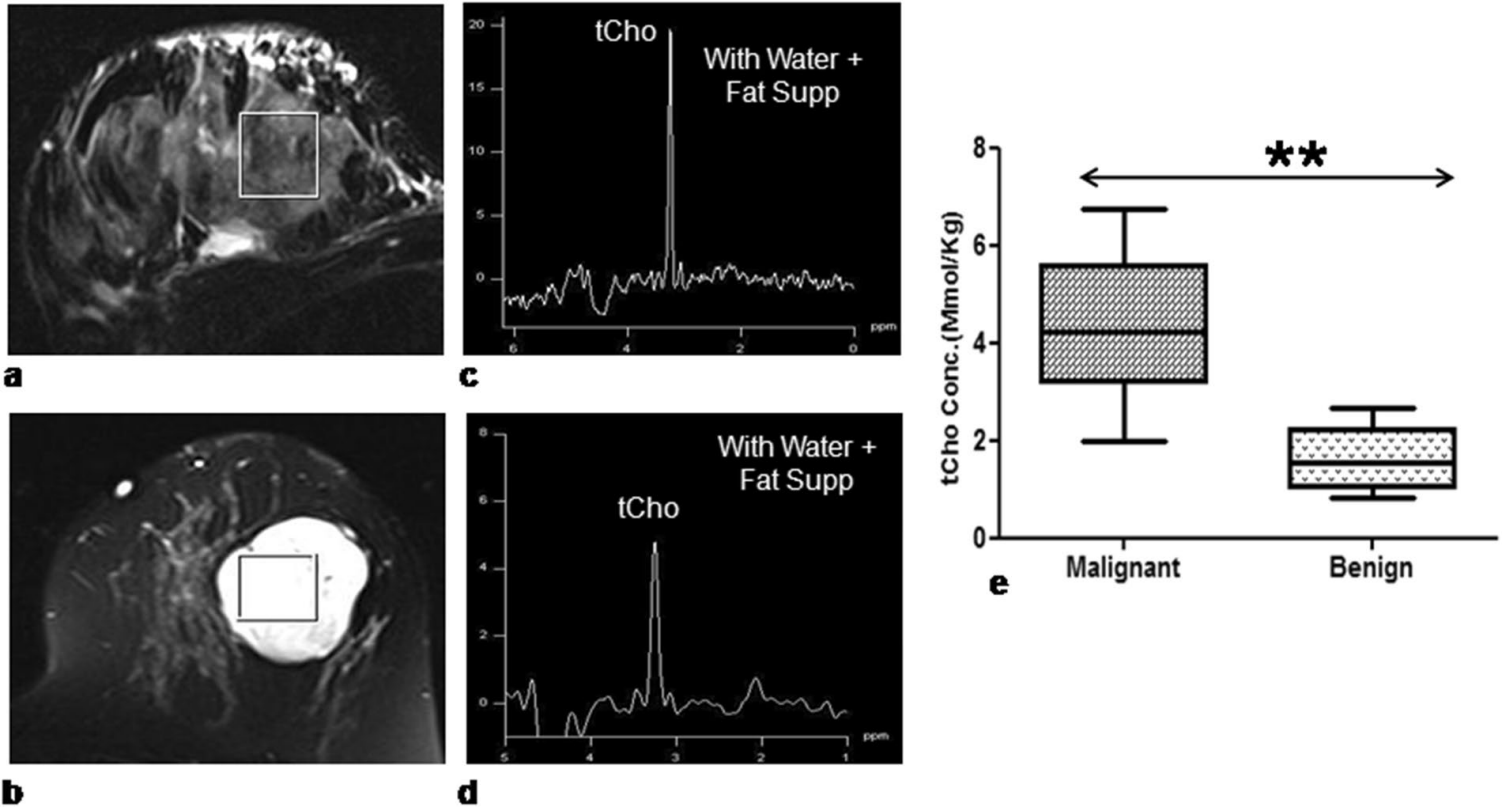
Representative example of T2 weighted fat suppressed axial image from a malignant and a benign lesion with the voxel location of size 8cc (20 × 20 × 20 mm) are shown as figures (**a**,**b**). The respective *in vivo* proton MR spectra with total choline (tCho) peak obtained with water and lipid suppression are shown as figures (**c**,**d**). While, the difference in tCho concentration (mmol/Kg) between malignant and benign breast tissues is shown as a box plot in figure (**e**).

The representative T2 weighted fat-saturated axial images of a patient with IDC and a patient suffering from fibroadenoma (benign), are shown in Fig. 2(a) and (b), respectively. Figure 2(c,d) are the respective ^1^H MR spectra acquired from voxels positioned in the lesion as shown in the images. Malignant tissues had significantly higher median (range) tCho concentration [4.2 (2.0–6.7) mmol/Kg] compared to benign tissues [1.6 (0.8–2.7) mmol/Kg] (Fig. 2e, Table 1).

**Table 1:** Distribution of median tCho, β-catenin and Cyclin D1 concentrations in malignant, benign and non-involved breast tissues.

Groups (n)	tCho (mmol/Kg) [Median (range)]	β-catenin (pg/ml) [Median (range)]		Cyclin D1 (ng/ml) [Median (range)]	
		Cytosolic fraction	Nuclear fraction	Cytosolic fraction	Nuclear fraction
Malignant tissues (n = 20)	4.2^#^ (2.0–6.7)	5.1*^#^ (2.3–7.5)	10.5*^#^ (3.0–39.7)	2.0*^#^ (1.0–2.3)	2.1*^#^ (2.0–2.7)
Benign tissues (n = 10)	1.6^#^ (0.8–2.7)	3.9^#^ (2.0–5.8)	5.7^#^ (0.5–17.6)	2.0^#^ (1.5–3.5)	2.0^#^ (2.0–2.4)
Non-involved tissues (n = 20)	@	4.0^#^ (2.0–5.9)	5.7^#^ (0.5–19.5)	2.0^#^ (2.0–3.0)	2.1^#^ (2.0–2.7)

Symbols *^#^denotes significance with p < 0.05 where, *difference in expression of protein in cytosolic and nuclear fractions within groups ^#^difference in tCho and expression of protein between groups ^@^Note: *In vivo* ^1^H MRS could not be carried out in non-involved tissues.

### Expression of β-catenin and Cyclin D1

**Figure 3:**
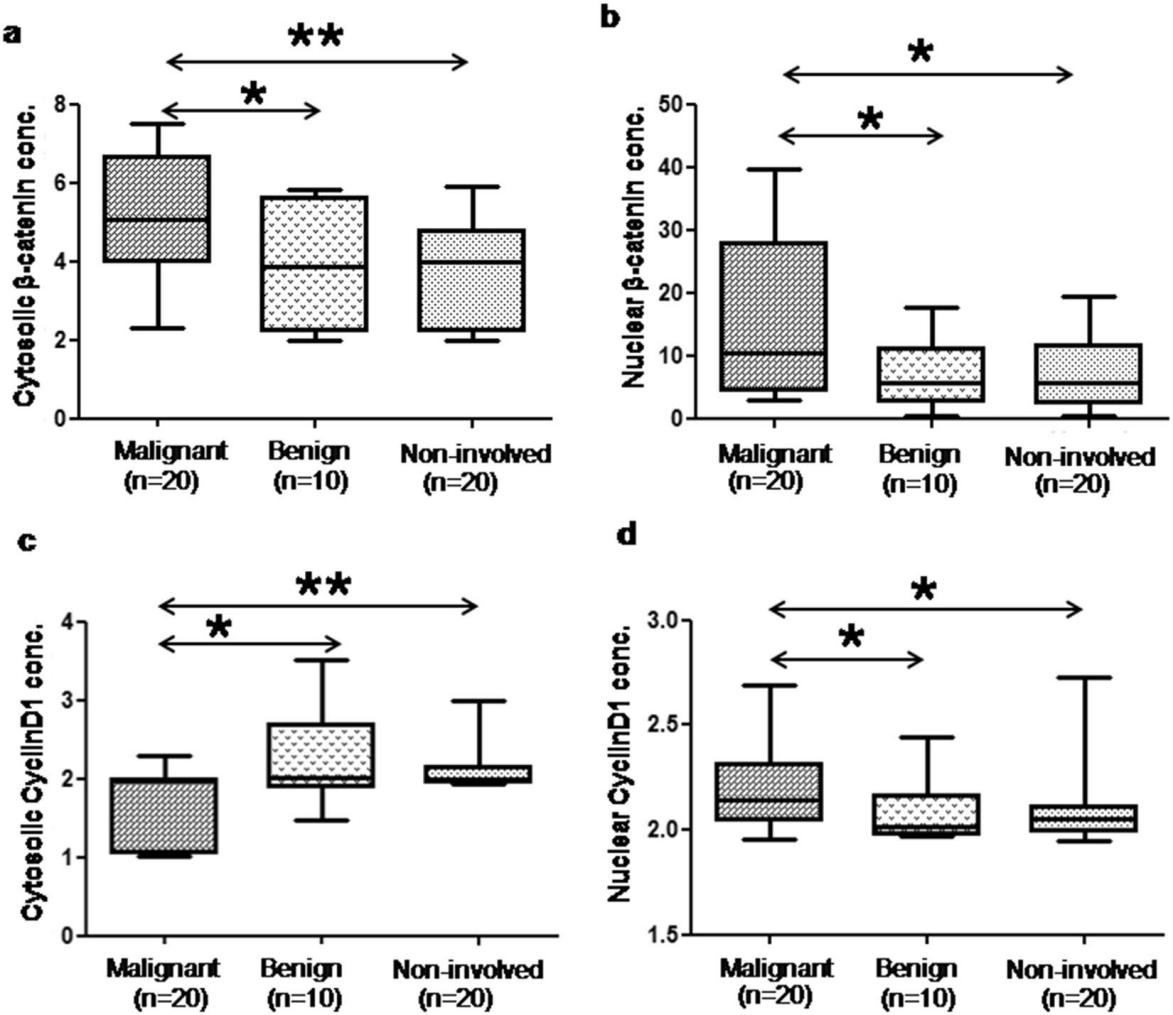
Box plots showing difference in (**a**,**c**) cytosolic β-catenin expression (pg/ml) and cyclin D1 expression (ng/ml) among malignant, benign and normal healthy breast tissues, (**b**,**d**) nuclear β-catenin expression (pg/ml) and cyclin D1 expression (ng/ml) in malignant, benign and normal healthy breast tissues obtained using ELISA. Here **denotes significance with p < 0.01 while, *denotes p < 0.05.

The expression of both cytosolic and nuclear β-catenin was significantly higher in malignant [5.1 (2.3–7.5) pg/ml; 10.5 (3.0–39.7) pg/ml] compared to benign [3.9 (2.0–5.8) pg/ml; 5.7 (0.5–17.6) pg/ml) and non-involved breast tissues [4.0 (2.0–5.9) pg/ml; 5.7 (0.5–19.5) pg/ml] (Fig. 3a and b, Table 1). The expression of β-catenin was significantly higher in the nucleus in comparison to the cytosol in malignant breast tissues (Table 1). The expression of cyclin D1 was studied in the same set of samples and it was found to be significantly overexpressed in nuclear fraction [2.1 (2.0–2.7) ng/ml] when compared to the cytosolic fraction [2.0 (1.0–2.3) ng/ml]. However, there was no significant difference in the expression of the protein in the cytosolic and the nuclear fraction of benign [2.0 (1.5–3.5) ng/ml; 2.0 (2.0–2.4) ng/ml] and non-involved breast tissues [2.0 (2.0–3.0) ng/ml; 2.1 (2.0–2.7) ng/ml] (Table 1). Also, the expression of cyclin D1 in cytosolic fraction was significantly higher in benign and non-involved breast tissues as compared to malignant tissues (Fig. 3c, Table 1). A significantly higher expression of cyclin D1 in the nuclear fraction of malignant tissues was seen in comparison with benign and non-involved breast tissues (Fig. 3d, Table 1). The expression of β-catenin and cyclin D1 in cytosolic and nuclear fractions did not show any significant difference between different tumor stages and menopausal status of breast cancer patients.

### Correlation analysis between β-catenin and cyclin D1 expressions and with total choline concentration

**Figure 4:**
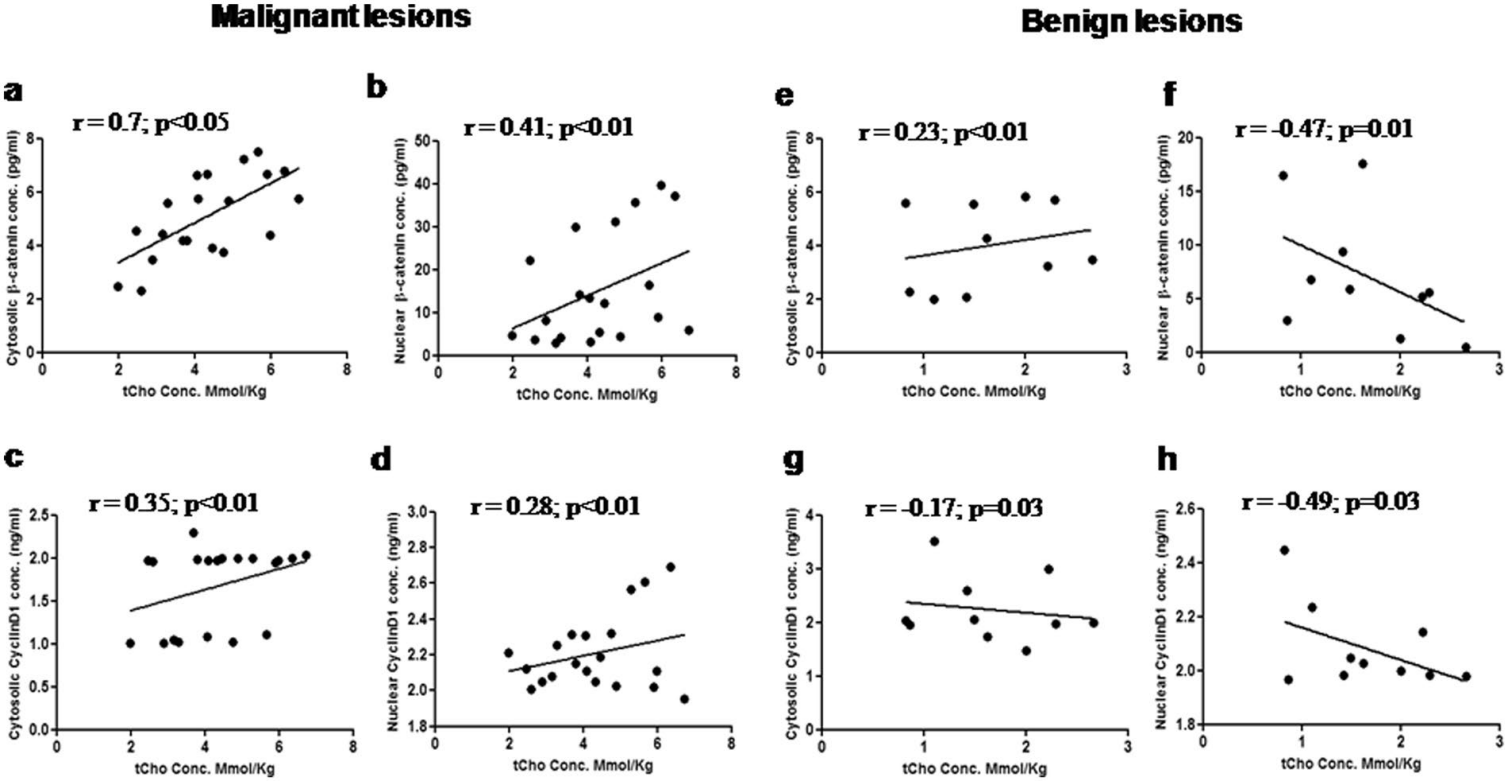
Scatter plots (**a**–**d**) represent the positive correlation of cytosolic and nuclear expression of β-catenin and cyclin D1 with tCho levels in malignant breast tissues. While, (**e**) shows a weak positive correlation of cytosolic β-catenin with tCho levels in benign breast tissues and (**f**–**h**) show negative correlation of nuclear β-catenin and cytosolic and nuclear cyclin D1 with tCho levels in benign breast tissues.

Further analysis showed a strong positive Pearson correlation (r = 0.6; p < 0.01) between the expressions of β-catenin and cyclin D1 in the nuclear fraction of malignant tissues (see Supplementary Fig. S1). A significant positive correlation was seen between the expression of tCho and cytosolic β-catenin expression of malignant tissues (Fig. 4a), while a weak positive correlation was observed with the nuclear expression of the protein (Fig. 4b). Benign tissues showed a weak positive correlation between tCho and cytosolic β-catenin expression (Fig. 4c) and a negative correlation with the nuclear expression (Fig. 4d). Both cytosolic and nuclear cyclin D1 expressions showed a significant positive correlation with tCho in malignant tissues (Fig. 4e and f). However, a negative correlation was seen between tCho and both cytosolic and nuclear cyclin D1 expression in benign tissues (Fig. 4g and h).

### β-catenin and Cyclin D1 expressions and hormone receptor status of breast cancer patients

**Figure 5:**
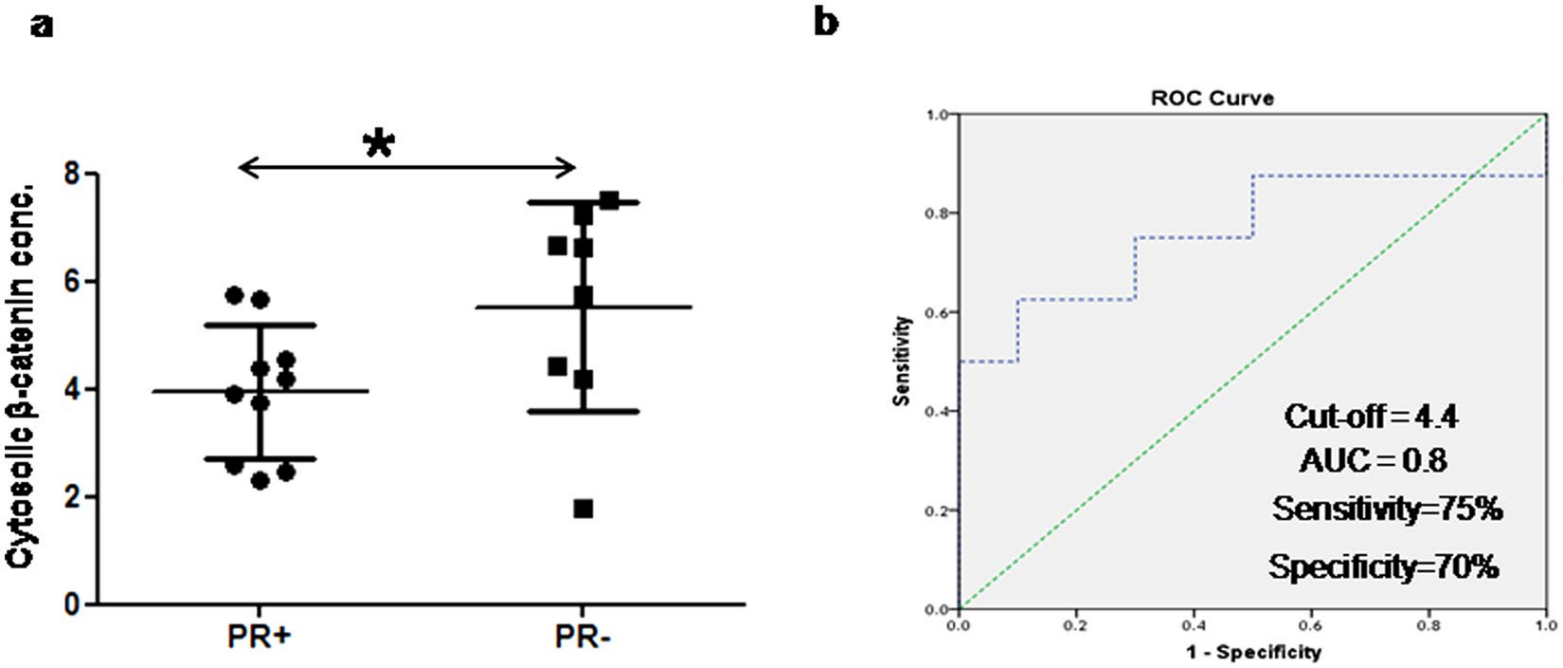
(**a**) Dot plot representation of the difference in cytosolic β-catenin expression (pg/ml) of progesterone receptor positive (PR+) and progesterone receptor negative (PR−) breast tumors obtained using ELISA. Here **denotes significance with p < 0.01 while, *denotes p < 0.05; (**b**) The receiver operator curve showing the cut-off of cytosolic β-catenin expression between PR− and PR+ breast tumors.

The expressions of β-catenin and cyclin D1 were also compared in breast cancer patients with different hormone receptor statuses. Cytosolic β-catenin expression was significantly higher in PR− [5.1 (1.8–7.5) pg/ml] compared to PR+[4.0 (2.3–5.8) pg/ml] breast cancers (Fig. 5a; Table 2). Receiver operator curve (ROC) analysis was carried out and a cut-off value of 4.4 pg/ml with a sensitivity and specificity of 75% and 70% was obtained for cytosolic β-catenin to discriminate between PR− and PR+ breast tumors (Fig. 5b). A significant positive association (r = 0.6; p < 0.05) was seen between PR and cytosolic β-catenin expression. There was no significant difference in β-catenin expression between patients with positive and negative ER and Her2neu receptor status. The expression of cyclin D1 was not significantly different in breast cancer patients with various hormone receptor groups.

**Table 2:** Distribution of median tCho, β-catenin and cyclin D1 concentrations in breast cancer patients (n = 20) based on the difference in their AJCC stage, menopausal status, and hormonal marker status.

Groups (n)	tCho (mmol/Kg) [Median (range)]	β-catenin (pg/ml) [Median (range)]		Cyclin D1 (ng/ml) [Median (range)]	
		Cytosolic fraction	Nuclear fraction	Cytosolic fraction	Nuclear fraction
AJCC Stage IIA (n = 11)	5.7 (2.1–17.9)	4.2 (1.8–7.5)	8.9 (3.0–37.2)	2.0 (1.0–2.0)	2.1 (2.0–2.7)
III (A + B) (n = 9)	4.7 (3.5–9.6)	5.6 (2.5–7.2)	12.2 (4.3–35.7)	2.0 (1.0–2.3)	2.2 (2.0–2.6)
Pre menopausal (n = 10)	5.8 (3.5–8.6)	5.1 (2.3–7.5)	14.9 (3.1–39.7)	2.0 (1.0–2.0)	2.2 (2.0–2.6)
Post menopausal (n = 10)	4.5 (2.1–17.9)	4.2 (1.8–6.7)	6.7 (3.0–37.2)	2.0 (1.0–2.3)	2.1 (2.0–2.7)
ER+ (n = 9)	5.1 (3.5–9.6)	4.2 (2.3–6.6)	13.3 (3.7–37.2)	2.0 (1.0–2.3)	2.3 (2.0–2.7)
ER− (n = 11)	4.9 (2.1–17.9)	4.5 (1.8–7.5)	8.9 (3.0–35.7)	2.0 (1.0–2.0)	2.1 (2.0–2.6)
PR+ (n = 10)	5.3 (3.5–9.6)	4.0 (2.3–5.8)*	17.1 (3.7–39.7)	2.0 (1.0–2.3)	2.2 (2.0–2.7)
PR− (n = 10)	5.4 (2.1–17.9)	5.1 (1.8–7.5)*	11.1 (3.0–35.7)	2.0 (1.0–2.0)	2.1 (2.0–2.6)
Her2neu+ (n = 9)	4.7 (2.1–9.6)	4.4 (2.5–7.5)	16.5 (3.0–39.7)	2.0 (1.0–2.3)	2.2 (2.0–2.6)
Her2neu− (n = 11)	5.9 (3.5–17.9)	5.6 (1.8–7.2)	8.9 (3.1–37.2)	2.0 (1.0–2.0)	2.1 (2.0–2.7)

*Denote significant difference in cytosolic β-catenin between PR− and PR+ tumors with p < 0.05.

## Discussion

To the best of our knowledge, this is the first study that investigated the association of two major proteins (β-catenin and cyclin D1) implicated in Wnt pathway with the tCho levels determined non-invasively using *in vivo* ^1^H MRS in malignant, benign and non-involved breast tissues. In this study, the tCho concentration seen in malignant tissues was above the cut-off value of 2.54 mmol/Kg reported in our previous study^25^ and was significantly higher compared to that seen in benign lesions. A significantly higher tCho concentration observed in malignant tissues may be ascribed to an increase in the membrane turn over associated with an increase in cellular replication rate during cancer proliferation^15^. The increase in tCho is mainly attributed to increase in intracellular PC and the activation of enzymes involved in choline synthesis^23, 24^. *In vitro* ^1^H NMR studies have shown the presence of various choline containing compounds such as phosphoethanolamine (PE), GPC, PC and free choline in breast tumor extracts and *in vivo* cultured cancer cells^32, 33^. However, in the present study, only a composite peak of choline compounds (tCho) (comprising of free choline, PC, GPC and PE) at 3.23 ppm was observed as MRS was carried out at a low field (1.5 T) and these peaks could not be resolved. PE is involved in the biosynthesis of phosphatidyl ethanolamine (PtdEtn) which is second most abundant membrane phospholipid after phosphatidyl choline (PtdCho) in eukaryotes in breast cancer biology^32–35^. In order to sustain stress conditions induced by cancer cells, metabolic pathways like glycolysis and fatty acid synthesis gets upregulated^36–38^ leading to enhanced synthesis of membrane phospholipids.

Apart from tCho, while assessing the cytosolic and nuclear β-catenin expressions, both were found to be significantly higher in malignant compared to benign and non-involved breast tissues. Previous studies have shown the cellular location of β-catenin to be dependent on the state of malignancy. While it is located in the cellular membranes of normal epithelial and non-invasive tumor cells; in malignant tissues, this protein is mostly located in cytosol and nucleus^39^. Extrapolating these literature findings to the results presented here, the observations of high nuclear and cytosolic β-catenin can be attributed to the multi-step process of EMT (Epithelial-mesenchymal transition) – a key event responsible for the conversion of benign tumors to their malignant and invasive counterparts^40^. During EMT, there is a loss in the expression of several membrane proteins involved in cellular adhesion, including β-catenin, which acts as a key component in the assembly of epithelial cell sheets^41, 42^. As a result, β-catenin translocates first to the cytoplasm which is later followed by its translocation to the nucleus^43^. This loss of cell-cell adhesion and cell-extracellular matrix interactions further contributes to the increased invasive potential of cancer cells^44^. Moreover, complementing our results there are multiple lines of evidence that suggest the role of β-catenin in breast cancer and its increased accumulation in the cytoplasm and/or, nucleus of the cell during malignancy^9, 11, 12, 39, 45–50^.

Our results showed that the expression of β-catenin in malignant tissues was significantly higher in the nucleus as compared to the cytosol. β-catenin is involved in diverse cellular processes like growth, differentiation, and transcription of Wnt-responsive genes^48, 49^. In the presence of a Wnt signal, GSK3β losses its ability to phosphorylate casein kinase-primed β-catenin for ubiquitin-mediated degradation. As a result, β-catenin accumulates in the cytosol and later translocates to the nucleus, binds to TCF and, activates the transcription of genes involved in cell proliferation like cyclin D1^47, 48, 51^.

Furthermore, our data showed that the expression of cyclin D1 was increased in the nucleus compared to the cytosol of malignant tissues with no significant difference in the expression of the protein in the cytosol and nucleus of benign and non-involved breast tissues. This indicates an increased localization of protein from the cytoplasm to the cell nucleus during malignancy. Cyclin D1 forms a complex with CDKs and hyperphosphorylates the retinoblastoma protein (Rb) which inturn losses its ability to bind to the E2F family of transcription factors. Activated E2F then promotes the transcription of various genes required for the transition of cells from G1 to S phase, thus promoting cellular proliferation^52^. Cyclin D1 is a protein shown to be overexpressed in approximately 50% of human breast cancers^13^. It is reported that the gene encoding cyclin D1 gets overamplified during breast cancer and is predominantly localized in the nucleus of asynchronously dividing breast cancer cells^53, 54^. Lin *et al*. have shown β-catenin to regulate the expression of cyclin D1 in breast cancer cells^47^. They also demonstrated that the increased expression of cyclin D1 might be caused by the activation of β-catenin in breast cancer. Consistent with these findings, a strong positive correlation between the β-catenin and cyclin D1 expressions in the nuclear fraction of malignant tissue provides an evidence to the fact that the overexpression of β-catenin levels is associated with the upregulation of cyclin D1 levels during malignancy^10, 47, 55, 56^. Furthermore, an increased expression of cyclin D1 in the nucleus of malignant tissues in comparison to benign and non-involved breast tissues reflects the increased sequestration of cyclin D1 in the nucleus of highly proliferative cancerous cells.

Further, a positive correlation between the tCho levels and cytosolic β-catenin expression and a weak positive correlation with the nuclear expression of the protein in the malignant tissues was seen. It has been reported that PC is a major contributor to an increased tCho level observed in malignant breast tissues^24, 25^. The synthesis of PtdCho and PtdEtn, was first described in Kennedy pathway^27, 57^. In short, during PtdCho biosynthesis, the free choline is phosphorylated to PC by CHK^27, 57^ which is then converted to PtdCho by CCT^58^. PLD hydrolyzes PtdCho to Cho and phosphatidic acid during catabolism of PtdCho and the free Cho thus formed is again utilized for PtdCho synthesis. Thus, PC is both a precursor and a breakdown product of PtdCho and increase in its level may be attributed to an increase in the activities of enzymes implicated in both anabolic and catabolic pathways. Moreover, a study on colon cancer showed increased expression of β-catenin to be associated with an increase in the enzymatic activity of PLD; thus linking the catabolic PtdCho pathway with the Wnt/β-catenin signalling^31^. Hence, in breast malignancies as well, it is possible to envision a scenario where the translocation of β-catenin to cytoplasm contributes to an increased PLD activity, which leads to an increase in PC that contributes to the elevated tCho concentration observed.

Our data also indicated that in malignant tissues the increase of cyclin D1 levels is directly associated with increase in tCho levels. As discussed earlier, the increased activities of enzymes like PLD and CHK leads to enhanced tCho levels in malignant tissues. It has also been demonstrated in a study carried out on mammary human epithelial cell lines that CHK is an important molecule in regulating cell cycle mediators like cyclin D3^59^.

Further, an increased expression of cytosolic β-catenin was seen in PR− compared to PR+ breast tumors. A cut-off value of 4.4 pg/ml for cytosolic β-catenin with a sensitivity and specificity of 75% and 70% to discriminate between PR− and PR+ breast tumors was detected. Lin *et al*., in a study carried out on human breast cancer cell line showed decreased β-catenin expression in cells treated with progesterone^60^. Immunohistochemical studies on human endometrial cell lines showed that in the absence of progesterone in PR− tumors, the Wnt/β-catenin signalling is inhibited leading to the growth in cancer cells^61^. Therefore, the β-catenin expression is expected to increase in the cytosol of PR− breast tumors.

There are certain limitations of this study. Firstly, it has limited number of samples in malignant and benign category. Second, the study also needs to be conducted in a large cohort to substantiate the findings of the change in expression of these proteins with different hormonal receptor status of breast cancer patients. Nevertheless, the preliminary findings of the present study open up avenues for further study on the molecular pathways involved in breast cancer which would enable us to understand the biology of cancer progression. Third, the expression of proteins needs to be carried out in various subtypes of breast cancer. In the present study there were 18 invasive ductal carcinoma (IDC), but other subtypes were few [1 IDC+ ductal carcinoma *in situ* (DCIS) and 1 invasive carcinoma] and hence comparison between various subtypes could not be done. Lastly, high resolution magic angle spinning (HRMAS) of these tissues may provide information on the levels of PC, GPC and free choline. However, such a study could not be carried out due to lack of such a facility.

In conclusion this study demonstrated the role played by tCho, β-catenin, and cyclin D1 in cancer cell development and proliferation. This interesting finding is the first evidence, to our knowledge, which provided a link between choline synthesis and Wnt-regulated β-catenin pathways in human breast cancer. This may aid in understanding the changes in the signalling network that occur during breast malignancy. The data further lend credence to the fact that the enhancement of tCho during malignancy may be attributed to the translocation of β-catenin from cytosol to the nucleus of the cell. Further, the increased expression of cytosolic β-catenin expression in PR− tumors indicate the regulation of β-catenin signalling by progesterone during the development of breast cancer. We anticipate that these results will complement the existing understanding of enhanced tCho levels during breast malignancy. Future studies on the details of these molecular pathways could help in the identification of potential targets for the development of novel therapeutic strategies.

## Patients and Methods

### Patients

A total of 100 fractions (50 cytosolic and 50 nuclear fractions) were isolated from tissues collected from 20 malignant (IDC), 10 benign and 20 non-involved breast tissues. The subjects were recruited from the outpatient department of surgical disciplines of our Institute between the years 2013 to 2015. The age of the patients ranged from 26–65 years with a median age of 49 years. The clinicopathological parameters are summarized in Table 3. The surgical breast tissue specimens (50–100 mg) of the same subjects were collected immediately after surgery and stored at −80 °C until further use. None had received radiotherapy, chemotherapy or steroid hormone medications prior to surgery. Non-involved breast tissues (n = 20) were collected from a distance of 1 cm away from the tumor margin for IDC patients^62^. The institutional ethical committee approved the study, and written informed consents were obtained from each subject. All methods were carried out in accordance with relevant guidelines and regulations. The experimental protocols were approved by the institutional committee.

**Table 3:** Clinical and pathological characteristics of breast cancer patients.

Total number of patients (n = 20)	
Age in years [mean ± SD (range)]	48.4 ± 11.5 (26–65)
**AJCC stage at diagnosis**	**Number**
IIA	11
IIIA	4
IIIB	5
**Tumor size** (**Median and range in cm**)	4 (1.5–12)
**Histological type**	
Infiltrating ductal carcinoma (IDC)	18
Infiltrating ductal carcinoma with ductal carcinoma *in situ* (IDC + DCIS)	1
Invasive carcinoma	1
**Hormone Receptor Status**	
ER+	9
ER−	11
PR+	10
PR−	10
Her2neu+	9
Her2neu−	11

### Histopathology and TNM Staging

The surgically resected tumor specimens were fixed with 10% buffered formalin, embedded in paraffin, and stained with hematoxylin and eosin for pathological study, including histological typing. ER, PR, and Her2neu expression statuses were determined using the standard immunohistochemistry (IHC) protocol of routine clinical diagnosis followed in our institute. Patients were characterized into different hormone receptor types i.e., ER+, ER−, PR+, PR−, Her2neu+, and Her2neu−, based on the presence or absence of the expression of these receptors in tumors. American Joint Committee on cancer TNM staging criteria were followed for the clinical staging of breast cancer.

### *In vivo*^1^H Magnetic Resonance Spectroscopy

MR investigations were performed using a four-channel phased array receive breast matrix coil at 1.5 T (Avanto, Siemens, Healthcare, Germany). Patients were positioned head first prone with both the breasts fitting into the cups of the breast coil. MR studies were carried out at least after a week of core biopsy so as to allow inflammation or edema to settle down. Following the scout image, short inversion recovery coronal images were acquired (TR/TE = 6940/58 ms; slice thickness = 3 mm; and matrix size = 320 × 256). Also, fat-suppressed MR images were acquired in the transverse and sagittal planes with the following parameters: TR/TE = 6270/102 ms; slice thickness 3 mm with no gap; and matrix size = 512 × 440.

A single voxel *in vivo* ^1^H MRS was carried out using point-resolved spin-echo (PRESS) sequence^63^ with TR/TE = 1500/100 ms; NSA = 128; spectral width = 1000 Hz; vector size = 1024. For MRS data acquisition, a single voxel (range of 10 × 10 × 10 to 10 × 35 × 45 mm^3^ depending on the tumor size) was placed on the reference MR images by carefully avoiding the necrotic areas of the tumor. *In vivo* ^1^H MRS was carried out by localizing the voxel on the visible tumor carefully avoiding the necrotic areas using the T2W Fat Sat (fat saturated) and diffusion weighted images^64^. The restricted hypointense appearing viable areas and the hyperintense areas with dead cells could be well differentiated on a lesion using apparent diffusion coefficient (ADC) maps which helps in proper voxel positiong during MRS acquisition by avoiding the dead necrotic areas. The tumor volume ranged from 5.1 to 138.0 cm^3^ for patients recruited in this study, while size of the voxel used for MRS acquisition ranged from 1 to 15.8 cm^3^. This ensured that the size of the voxel for MRS was smaller than the tumor volume as well within the tumor. Magnetic field shimming was carried out both globally and over the voxel prior to MRS. The line-width at half maximum after voxel shimming corresponded typically to 5–15 Hz for the water peak in the spectra obtained from tumors. Water suppression was carried out using a frequency-selective presaturation pulse^65^ with a bandwidth of 50 Hz. For lipid suppression, a bandwidth of 1.8 ppm with the start and end frequencies for the fat region set at 0.4 and 2.2 ppm was used. From the same voxel position, an additional spectrum without water and lipid suppression with following parameters was acquired: TR = 2000 ms, TE = 100 ms, and a number of average = 1. Post-processing of an acquired spectrum was carried out using Syngo GRACE software provided by the manufacturer. For spectral post-processing 2.0-Hz line broadening with a polynomial order 5 baseline correction was used^25^. Automated normalization of the peak areas with internal water reference signal was done and normalized integral values (area under the curve) of the peaks were determined. The tCho concentration was then calculated using the formula mentioned in our previous reports^25^.

### Isolation of Cytoplasmic and Nuclear Protein Fractions

Isolation of protein fractions was carried out as per the methodology followed by Pu *et al*.^66^. Breast tissues (50–100 mg) were cut into small pieces and ice-cold Cytosol buffer (0.08% potassium chloride, 0.02% magnesium chloride, 0.3% HEPES, 006% NP-40 and 0.005% EDTA) was immediately added to cover the tissue completely. Sonication was performed for 20 minutes with the tube placed in ice to avoid heating of the sample. The sample was then centrifuged at 13,000 g for 15 minutes at 4 °C, and the lipid layer from the top was removed. Followed by a repeated centrifugation at 13,000 g for 15 minutes, the supernatant (cytosol-protein enriched) was carefully collected into a clean storage tube and the pellet retained. Wash buffer (0.08% potassium chloride, 0.02% magnesium chloride, 0.3% HEPES and 0.005% EDTA) was added to the pellet obtained. The lysate was vortexed and centrifuged at 3,000 g for 5 minutes at 4 °C. The supernatant was discarded and the nuclear buffer (4% sodium chloride, 0.02% magnesium chloride, 0.5% HEPES, 30% glycerol and 0.009% EDTA) was added. The lysate was incubated on ice for 30 min and vortexed every 10 min. The mixture was centrifuged at 13,000 g for 20 min at 4 °C. The supernatant enriched in nuclear-protein was carefully collected and transferred to a clean storage tube. The extracted cytoplasmic and nuclear protein fractions were stored at −80 °C.

### Western Blot

Isolated protein fractions were quantified by the Bradford method (Bio-Rad Laboratories) with bovine serum albumin as a standard (Bradford assay). To check the purity of the isolated fractions, glyceraldehyde 3-phosphate dehydrogenase (GAPDH) and Histone H2b were used as cytosolic and nuclear loading protein controls, respectively. Samples each from cytosolic and nuclear fractions extracted from malignant, benign and non-involved breast tissues were randomly chosen for western blot analysis. Equal amounts of proteins (20 μg/lane) were electrophoresed in 12% sodium dodecyl sulfate-polyacrylamide gels and transferred onto nitrocellulose membrane. After transfer, the blot was blocked with 4% bovine BSA in Tris-buffered saline and blots were incubated with the anti-GAPDH antibody (polyclonal anti-human GAPDH, Abcam; 1:500 dilution), anti-Histone H2b antibody (polyclonal Histone H2b antibody, Elabsciences; 1:500 dilution), at 4 °C overnight. Membranes were incubated with secondary antibody, HRP-conjugated rabbit anti-IgG (Abcam) (1:10,000) in 1% BSA for 2 hrs at room temperature. Protein bands were visualized using an enhanced chemiluminescence system (ECL, Santa Cruz Biotechnology, CA; see Supplementary Fig. S2).

### Enzyme Linked Immunosorbent Assay (ELISA)

ELISA experiments were performed using standard kits (β-catenin; SEB021Hu, Cyclin D1; SEA585Hu, Cloud-Clone Corp. Houston, USA) and the assay procedure were followed as per the instructions in the manual. Following surgery, 50–100 mg of the excised tumor tissues obtained were used for total protein isolation which were then quantified and used for ELISA experiments.

Different dilutions of the standards (as per manual instructions) and the samples were prepared and added to the ELISA plate coated with a mouse monoclonal antibody. Following the incubation and washing steps, rabbit polyclonal antibody (biotin conjugated), and avidin conjugated to HRP were added. The color change (blue color) at the end of reaction was observed by adding a substrate solution. The reaction was then stopped using stop solution provided in the kit so that the color changed to yellow. The absorbance values were then obtained on microplate reader (Epoch Microplate Spectrophotometer; Winooski, VT, USA) at 450 nm. Standardization was done in a manner that standard curves had a R^2^ value of 0.9.

To normalize the obtained absorbance data, duplicate readings for the samples were averaged and subtracted with the average zero standard reading. The concentrations of proteins in different fractions of the breast tissues were then reported in terms of pg/ml (β-catenin) and ng/ml (Cyclin D1). Intra-assay variability was <5%, whereas inter-assay variability was <10%.

### Statistical Analyses

Data in the tables are presented as median and range due to the high standard deviation. Student’s unpaired t-test (two-tailed) and Mann-Whitney were performed for determining the statistical significance of the tCho and protein concentrations in different breast tissues. Wilcoxon matched pairs two-tailed test was used to calculate the association between clinicopathological parameters and protein expression. The significant value of each analysis was defined at p < 0.05. Pearson correlation analysis was carried out to determine the correlation between tCho and protein (β-catenin and Cyclin D1) concentrations in different tissue lysates. All the statistical analysis was performed using SPSS 16.0 for Windows (SPSS Inc., Chicago, IL).

## Electronic supplementary material

Supplementary information
