# Supplementary material for: Is there an association between enhanced choline and β-catenin pathway in breast cancer? A pilot study by MR Spectroscopy and ELISA
Source: Sci Rep. 2017 May 22;7:2221. doi: 10.1038/s41598-017-01459-z (PMC5440410; doi:10.1038/s41598-017-01459-z)
Supplement: Supplementary file 1 — Supplementary information [file 41598_2017_1459_MOESM1_ESM.pdf]

Is there an association between enhanced choline and β-catenin pathway in breast cancer? A pilot study by MR Spectroscopy and ELISA

Khushbu Agarwal[1], Gururao Hariprasad[2], Komal Rani[2], Uma Sharma[1*], Sandeep R. Mathur[3], Vurthaluru Seenu[4], Rajinder Parshad[4] and Naranamangalam R. Jagannathan[1*]

Departments of NMR & MRI Facility[1], Biophysics[2],

Pathology[3] and Surgical Disciplines[4]

All India Institute of Medical Sciences

New Delhi – 110029, India

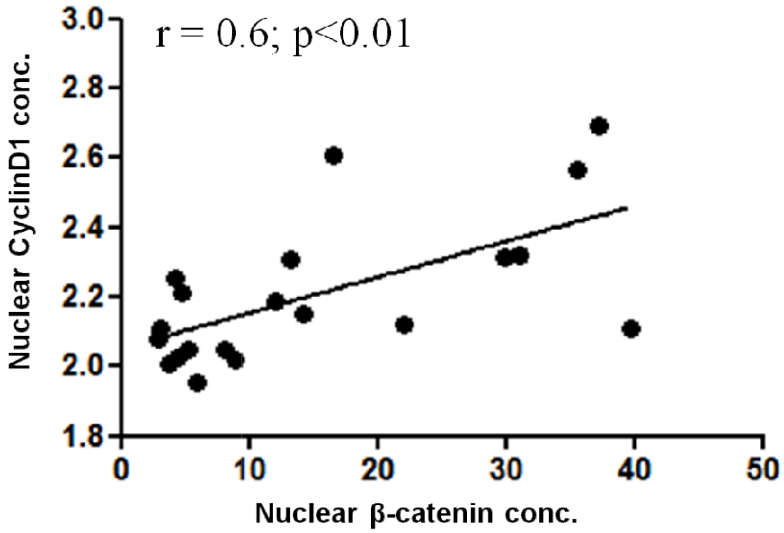

Supplementary Fig S1. Scatter plot showing positive correlation between nuclear expression of β-catenin (pg/ml) and Cyclin D1 (ng/ml) in malignant breast tissues

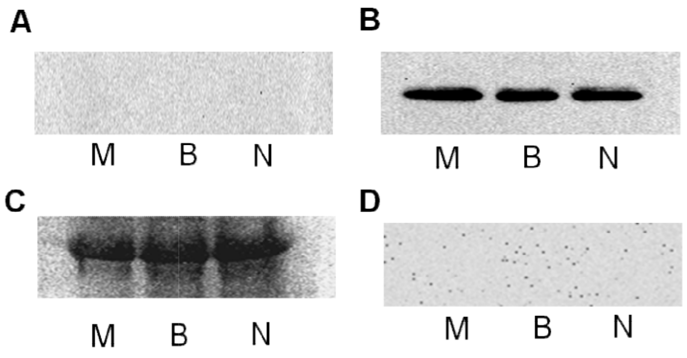

Supplementary Fig S2. Western blot of cytosolic and nuclear breast tissue lysates, here (A) & (D) represents the lack of nuclear and cytosolic protein contamination; (B) shows the band of HistoneH2b in nuclear fraction in malignant, benign and healthy normal breast tissues and; (C) shows the band of GAPDH in cytosolic fraction of malignant, benign and healthy normal breast tissues.
